# Cutaneous manifestations and treatment of arsenic toxicity: A systematic review

**DOI:** 10.1002/ski2.231

**Published:** 2023-03-25

**Authors:** Gia Toan Tang, Joshua Elakis, Laura Scardamaglia

**Affiliations:** ^1^ Department of Dermatology University of Melbourne Footscray Hospital Melbourne Victoria Australia; ^2^ Department of Dermatology Deakin University University Hospital Geelong Geelong Victoria Australia

## Abstract

Cutaneous and systemic signs of acute and chronic arsenic poisoning may be vague. Thus, an awareness of these signs is crucial to prevent late or missed diagnoses. This is especially true in non‐endemic countries where individuals may present decades after exposure, or may still be ingesting arsenic via a non‐classical exposure. Existing literature emphasizes several well‐known cutaneous presentations of arsenic toxicity while ignoring the complete clinical spectrum, including several rare tumours of relevance to the dermatologist. This study aims to review the existing literature on dermatological presentations of arsenic toxicity and their management in adults.

## Graphical abstract

This article reviews the clinical spectrum of malignant and non‐malignant presentations of chronic arsenic toxicity.

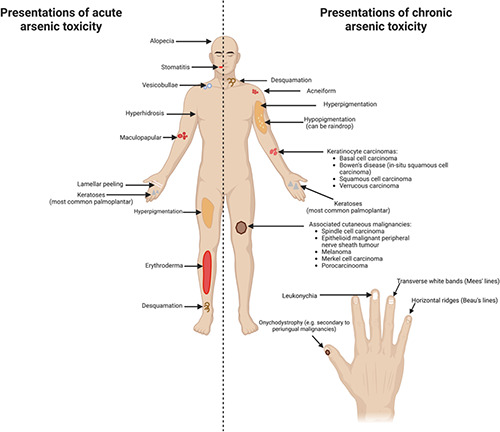

1

**What is already known about this topic?**

- Cutaneous arsenic toxicity has well‐known presentations such as hyperkeratosis and rain‐drop hyperpigmentation.
- It commonly occurs in non‐endemic countries where individuals have chronic exposure but may also be present in developed countries where there is exposure from occupational exposure and medicinal ingestion.

**What does this study add?**

- This review demonstrates that there is a broad spectrum of non‐malignant and malignant cutaneous presentations that are associated with chronic arsenic exposure but are often not recognized.
- However, given limited studies in current literature, associations are still weak but should still be considered given that chronic arsenic toxicity are often missed or underdiagnosed.

## 2 INTRODUCTION

Arsenic is a naturally occurring carcinogenic heavy metal, often found in drinking water sources tapped from tube wells.^1^ The skin is susceptible to arsenic, with chronic exposure occurring from environmental, occupational, dietary and medicinal sources. The World Health Organization provisional guideline suggests arsenic level in drinking‐water to be less than 10 μg/L. However, in India and Bangladesh where regions of arsenic contamination are greatly affected, the permissible level recommended is less than 50 μg/L.^2^, ^3^ Arsenic has previously been used as a treatment for various conditions such as epilepsy, asthma, warts, psoriasis and syphilis.^4^ It can also be found in Chinese and Ayurvedic medicines. Arsenic may be absorbed via alimentary, respiratory, and transcutaneous routes leading to severe acute illness, hospitalization and death.^5^ Chronic arsenic toxicity occurs in individuals exposed to lesser quantities over longer periods of time (e.g., ingesting contaminated well water, occupational exposure to arsenic‐containing pesticides). In chronic exposure, arsenic accumulates in various organs, concentrating in ectodermal tissues including skin, hair and nails.^5^, ^6^

Acute arsenic toxicity results in capillary damage leading to oedema, hypoperfusion and shock. This is secondary to its effect of inhibiting enzymes of the Krebs cycle that subsequently impairs oxidative phosphorylation.^7^ Chronic exposure to arsenic may cause immunosuppression by inducing keratinocyte apoptosis through Fas/Fas‐ligand interaction, decreasing the percentage of cluster of differentiation 4 (CD4+) T cells in the peripheral blood, decreasing the numbers of Langerhans cells and altering their migration.^8^, ^9^ Its carcinogenic effect also causes chromosomal instability through hypo‐ and hypermethylation of DNA, amplification of genes and induction of sister‐chromatid exchanges.^8^, ^9^

Cutaneous presentations of arsenic toxicity have been widely reported in literature. Clinical suspicion of chronic arsenic exposure may involve the presence of multifocal non‐melanocytic skin cancers such as basal cell carcinomas (BCC), squamous cell carcinoma (SCC) and in‐situ SCC (Bowen's disease, BD) in sun‐exposed and sun‐protected skin.^10^, ^11^, ^12^, ^13^ To date, there are no systematic reviews to summarize and critically appraise reported studies of common as well as rare dermatological findings and their treatments. Subsequently, this article systematically aims to identify all dermatological manifestations, mode of exposure and treatments in adults with suspected arsenic exposure reported in current literature and assess the studies reporting these findings.

## 3 METHODS

### 3.1 Search strategy

A systematic review was conducted following the Preferred Reporting Items for Systematic Reviews and Meta‐Analyses (PRISMA) guidelines. A search of EMBASE via OVID and Medline via OVID database for articles from 1946 to August 2020 for terms in abstracts, titles, all fields and subject headings. No language, date or other limits were applied. Full search strategies are given in Table 1. The databases were deduplicated in Ovid then uploaded to Covidence, a web‐based software for further deduplication, screening, and full‐text evaluation using inclusion and exclusion criteria.

**TABLE 1:** Summary of cutaneous manifestations of acute arsenic toxicity.

Authors	LOE	Study design	Number of cases	Type of exposure	Non‐malignant cutaneous manifestations	Sites affected	Treatment of cutaneous presentations
Tay et al.,^14^ 1974	III	Retrospective cohort	*n* = 22	Medicinal	Erythroderma	Palmoplantar	Dimercaprol
					Hyperhidrosis		
					Exfoliation		
Uede et al.,^15^ 2003	IV	Prospective cohort (without comparator group)	*n* = 62	Dietary	Cracking	Scalp	Not reported
					Peeling	Lips	
					Erythema	Palmoplantar	
					Maculopapular	Chest	
					Keratosis	Abdomen	
					Lamellar peeling	Buttocks	
					Alopecia	Upper limb extremities	
					Stomatitis	Lower limb extremities	

Abbreviation: LOE, level of evidence.

### 3.2 Selection criteria

The protocol was registered with the international prospective register of systematic reviews (PROSPERO), in accordance with PRISMA guidelines (PROSPERO CRD42020203145). Candidate studies were selected by two independent authors (Dr GT and Dr JE) on Covidence on basis of titles and abstracts, then were obtained and read in full. The exclusion criteria were as follows: full‐text article inaccessible; animal‐based study; in vitro study; focussed on arsenic as a therapeutic treatment; non‐dermatological focus; uncertain causality or other causal of cutaneous presentation; non‐primary research article. Paediatric cases were initially part of the inclusion criteria for PROSPERO however was later in the exclusion criteria as authors agreed for the population study to focus on adults. The two authors agreed on the final selection of studies and discrepancies were solved by discussion.

### 3.3 Data extraction and critical appraisal

Data were collected through Microsoft Excel to capture data items and for data synthesis on the following: study design, recruitment method, sample size, types of exposure, country of exposure, non‐malignant and malignant cutaneous presentations reported as well as treatments. Each study was assigned a level of evidence according to the 2011 Oxford Centre for Evidence‐Based Medicine levels of evidence guidelines. Assessment of risk of bias for each selected study was performed by Dr GT and Dr JE according to the JBI checklist for bias for the corresponding study types and consensus was reached through discussion.

### 3.4 Synthesis of findings

Descriptive statistical analysis summarizing the consensus response for checklist questions for each study type was conducted. As identified studies were heterogeneous, meta‐analysis could not be conducted. A narrative synthesis was used as pooling of data is not possible due to the various modalities, presentations, treatment options and permutation of findings of arsenic toxicity reported in current literature. The criteria used by authors of this review to associate clinical features with arsenic toxicity in selected articles was if cutaneous presentations mentioned in case reports and case series were also identified in case‐control, retrospective and cross‐sectional studies. Associations were made based on the clinical presentations and the history provided in the articles suggestive of arsenic exposure or if there were also quantitative measurement of arsenic levels in suspected source or samples from patients. The criteria to consider treatment as potential management for dermatological presentations of suspected arsenic toxicity was if treatment was mentioned to be used in at least three case reports, or was mentioned in case reports and its use was also identified in other case series or retrospective studies.

## 4 RESULTS

### 4.1 Selection of studies and critical appraisals

**FIGURE 1:**
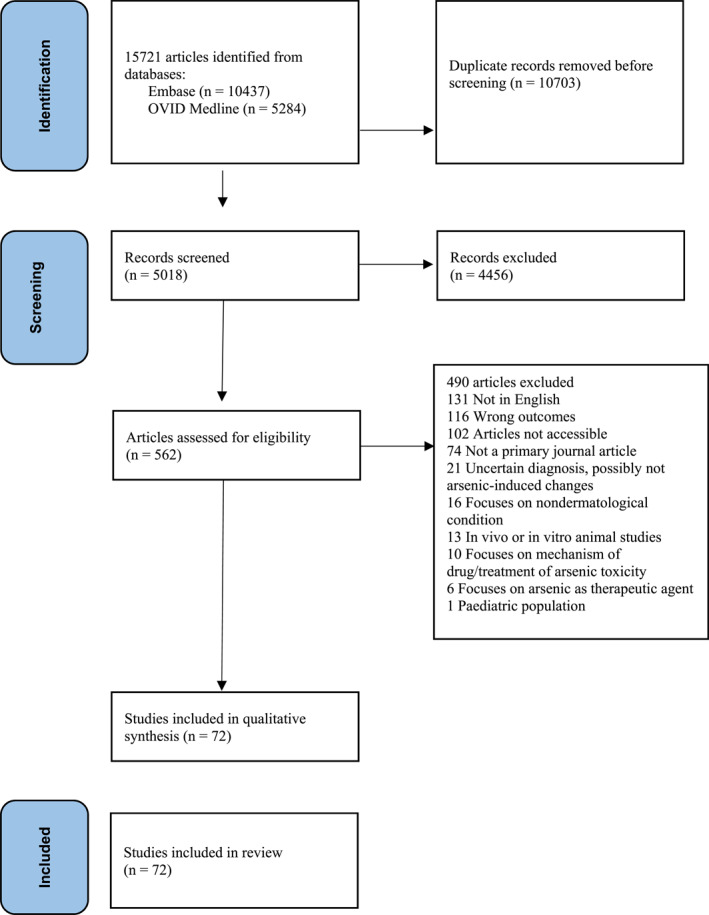
PRISMA diagram of literature search and selection of articles.

The search methods described identified a total of 15 721 articles from Embase and Medline through OVID, published from 1946 to 25 August 2020. After 10 703 duplicate records were removed, articles were screened based on title, abstract and full text as needed to determine eligibility. Retrieved studies were composed of 72 articles selected for review with case reports or series (*n* = 59), case‐control studies (*n* = 2), cross‐sectional studies (*n* = 9) and cohort studies (*n* = 2). Figure 1 summarizes the selection process. Tables 1, 2, 3 list the corresponding levels of evidence. Tables to summarize the JBI checklist consensus response for each study in risk of bias assessment were created, and the percentage of responses were calculated for each JBI checklist question domain for each study type as well as the overall appraisal (Supporting Information S1). After risk of bias assessment, two case reports had ‘Seek further information’ as overall appraisal, one for case series, one for case‐control, and one for cross‐sectional. However, no studies were excluded after risk of bias assessment as authors did not deem any studies having a high risk of bias warranting exclusion.

**TABLE 2:** Summary of cutaneous presentations of chronic arsenic toxicity through medicinal and dietary exposure.

Authors	LOE	Study design	Number of cases	Non‐malignant cutaneous manifestations	Malignant cutaneous manifestations	Sites affected	Treatment
Centeno et al.,^6^2002	IV	Case series	*n* = 3	Keratosis	BD SCC BCC	Breast Fingers Neck	Not reported
Chakraborti et al.,^41^2003	IV	Case series	*n* = 3	Hyperpigmentation Hyperkeratosis Hypopigmentation	Not reported	Upper limb extremities Lower limb extremities Trunk Plantar of foot Generalized	Discontinued exposure
Ding et al.,^17^ 2018	V	Case report	*n* = 1	Not reported	BD BCC	Upper limb Back	PDT
Gerdsen et al.,^18^ 2000	V	Case report	*n* = 1	Keratoses Hypopigmentation	Not reported	Palmoplantar Trunk Upper limb Lower limb	10% salicylic acid in white soft paraffin Cryotherapy 0.1% tazarotene gel
Hanjani et al.,^19^ 2007	V	Case report	*n* = 1	Keratosis Hyperpigmentation Hypopigmentation	Not reported	Palmoplantar Chest Back Abdomen	Discontinued exposure
Hill et al.,^20^ 1964	V	Case report	*n* = 1	Keratosis	SCC	Fingers Palmoplantar	Excision and skin graft
Hinojosa et al.,^21^ 2018	V	Case report	*n* = 1	Keratosis Hypopigmentation	Not reported	Generalized	Excision Nicotinamide (prevention)
Kaur et al.,^22^ 1982	V	Case report	*n* = 2	Keratosis Ulceration Hyperpigmentation Atrophy Erythema Hypopigmentation	BD	Fingers Generalized Trunk Palmoplantar	Not reported
Kim et al.,^23^ 1999	V	Case report	*n* = 1	Erythema	BD BCC	Hand	Cryotherapy
						Back	
Kim et al.,^24^ 2013	V	Case report	*n* = 1	Keratosis	SCC	Plantar surface of feet	Excision and skin graft
Lonergan et al.,^55^ 2010	V	Case report	*n* = 1	Keratosis	SCC	Upper limb Lower limb Trunk Palmoplantar	Moh's surgery 5% imiquimod cream
Murata et al.,^26^ 1994	V	Case report	*n* = 1	Keratosis	BD	Palmoplantar Chest	Resection
Park et al.,^63^ 2002	V	Case report	*n* = 1	Hyperpigmentation	BD	Palmoplantar Trunk	5‐Fluorouracil
Pinto et al.,^17^ 2014	V	Case report	*n* = 2	Raindrop pigmentation Keratosis Mees' lines	Not reported	Nails of the hands Palmoplantar Upper limb Lower limb Abdomen Back	Discontinued exposure D‐penicillamine
Pollo et al.,^29^ 2019	V	Case report	*n* = 1	Not reported	BCC	Nails of the hands	Excision and skin graft
Ramirez et al.,^38^ 2012	V	Case report	*n* = 1	Not reported	Porocarcinoma BD SCC	Toes Vulva	Not reported
Sass et al.,^27^ 1993	V	Case report	*n* = 1	Keratosis	SCC	Palmoplantar Fingers	Discontinued exposure
Sass et al.,^31^ 1993	V	Case report	*n* = 1	Hyperpigmentation Keratosis Mees' lines	SCC BD	Generalized Palmoplantar Nails of hand	Excision Acitretin Urea cream Salicylic acid 5‐Fluouracil
Seok et al.,^32^ 2015	V	Case report	*n* = 1	Erythema Keratosis	BD SCC	Palmoplantar Lower limb extremities	Imiquimod
Shneidman et al.,^10^ 1986	V	Case report	*n* = 1	Hyperkeratosis	Dermatofibroma protuberans BCC BD	Palmoplantar	Shave excision
Siefring et al.,^5^ 2018	V	Case report	*n* = 1	Alopecia Ulceration (oral and skin) Mees' lines Hypopigmentation	SCC	Scalp Fingers Mouth Palms of hands Upper limb extremities Perianal Lower limb extremities	Discontinued exposure Excisions Nicotinamide
Sommers et al.,^33^ 1953	IV	Case series	*n* = 17	Not reported	SCC BCC BD Hidradenocarcinoma	Palmoplantar Upper limb extremities Lower limb extremities Face Neck Breast Trunk Back Scrotum Vulva Anal	Excision and skin grafts Amputation Radiation therapy Radioactive ointment
Southwick et al.,^34^ 1979	V	Case report	*n* = 1	Keratoses	SCC	GroinPalmoplantar	Not reported
Tantikun et al.,^11^ 2000	III	Retrospective cohort	*n* = 4	Keratosis	BD SCC	Fingers Palmoplantar	Carbon dioxide laser, with 2% mupirocin ointment
Tay et al.,^16^ 1974	III	Retrospective cohort	*n* = 52	Alopecia Keratoses Mees' lines Hyperpigmentation Raindrop hypopigmentation Mucus membrane irritation Keratoderma	BCC SCC	Scalp Palmoplantar Nails of the hands Upper limb extremities Lower limb extremities Abdomen	5‐Fluorouracil Dimercaprol
Uede et al.,^14^ 2003	IV	Prospective cohort [without comparator group]	*n* = 21	Mees' lines Beau's lines Leukonychia Onychodystrophy Post‐inflammatory hyperpigmentation Acneiform eruption Desquamation	Not reported	Nails of the hands Acral sites Labia Unspecified areas	Not reported
Wagner et al.,^15^ 1979	V	Case report	*n* = 1	Keratosis Erythema	BCC SCC	Trunk Upper limb extremities Lower limb extremities	Hydrocortisone cream Curettage and electrodessication Cryosurgery 5‐Fluorouracil
Wong et al.,^71^ 1998	IV	Retrospective cohort	*n* = 14	Keratosis Hyperpigmentation Hypopigmentation	BD SCC Melanoma	Palmoplantar Upper limb extremities Lower limb extremities Chest Back Buttock Scrotum	Not reported
Wong et al.,^37^ 1998	IV	Case series	*n* = 3	Keratosis Hypopigmentation Hyperpigmentation	BD BCC SCC	Fingers Palmoplantar Upper limb extremities Lower limb extremities Buttock	Discontinued exposure Excision
Zhou et al.,^34^ 2015	V	Case report	*n* = 1	Mees' lines Erythema Keratosis Desquamation	Not reported	Nails of hands Back Unspecified areas	Sodium dimercapto‐sulfonate
Zhu et al.,^64^ 2012	V	Case report	*n* = 1	Hyperpigmentation	Verrucous carcinoma	Lower limb extremities	Not reported

Abbreviations: BCC, basal cell carcinoma; BD, Bowen's disease; LOE, level of evidence; PDT, photodynamic therapy; SCC, squamous cell carcinoma.

**TABLE 3:** Summary of cutaneous presentations of chronic arsenic toxicity through environmental, occupational exposure and multiple exposures.

Authors	LOE	Study design	Number of cases	Country of exposure	Non‐malignant cutaneous manifestations	Malignant cutaneous manifestations	Sites affected	Treatments
Ahmad et al.,^32^ 1999	IV	Cross‐sectional	*n* = 363	Bangladesh	Keratosis Hyperpigmentation	BD	Generalized Palmoplantar	Not reported
Ahsan et al.,^43^ 2009	V	Case report	*n* = 2	Pakistan	Keratosis Hypopigmentation Hyperpigmentation	Not reported	Neck Palmoplantar Trunk	Discontinued exposure
Cabrera et al.,^42^ 2003	III	Retrospective	*n* = 23	Argentina	Keratosis Keratoderma Hyperpigmentation	BD BCC SCC	Face Back Axilla Upperlimb extremities Trunk Vulva Plantar surface of feet	Not reported
Chen et al.,^9^ 2005	V	Case report	*n* = 1	Taiwan	Keratosis Hyperpigmentation	BD Porocarcinoma	Chest Palmoplantar	Surgical excision and flap repair
Chen et al.,^43^ 2017	V	Case report	*n* = 1	Taiwan	Keratosis	BD EMPNST	Chest Back Hip	Surgical excision with skin graft
Chou et al.,^8^ 2016	V	Case report	*n* = 1	Taiwan	Not reported	BD SCC	Breast	Cryotherapy
Choudhury et al.,^12^ 2018	III	Retrospective	*n* = 960	Bangladesh	Not reported	BCC SCC Melanoma MCC	Face Scalp Upper limb extremities Lower limb extremities Trunk	Surgical excision with graft/flap
Col et al.,^44^ 1999	V	Case report	*n* = 1	Turkey	Keratosis	BD Spindle cell carcinoma	Palmoplantar Lower limb extremities	Surgical excision with skin graft
Das et al.,^44^ 2012	V	Case report	*n* = 1	India	Alopecia Hypopigmentation Hyperpigmentation Keratosis	Not reported	Scalp Palmoplantar Chest	Discontinued exposure D‐penicillamine
Ghosh et al.,^18^ 2013	IV	Case‐control	*n* = 73	India	Keratosis Raindrop hyperpigmentation	Not reported	Palmoplantar	Not reported
Ghosh et al.,^72^ 2013	IV	Case series	*n* = 24	India	Alopecia Keratosis Hyperpigmentation	BCC BD SCC	Scalp Face Fingers Palmoplantar Upper limb extremities Neck Chest Lower limb extremities Trunk Buttock	Imiquimod Surgical excision
Gulshan et al.,^48^ 2016	V	Case report	*n* = 1	India	Hyperpigmentation	BCC	Scalp Trunk	Discontinued exposure
Ho et al.,^49^ 2005	V	Case report	*n* = 2	Taiwan	Keratosis Raindrop hypopigmentation	BD MCC	Scalp Breast Abdomen	Surgical excision with/without skin graft Radiotherapy
Ishinishi et al.,^50^ 1977	IV	Cross‐sectional	*n* = 9	Japan	Hyperkeratosis Hyperpigmentation Hypopigmentation Alopecia	Not reported	Scalp Palmoplantar Neck Upper limb extremities Lower limb extremities Chest Back Abdomen	Not reported
Jaafar et al.,^52^ 1993	IV	Case series	*n* = 3	Malaysia	Keratosis Hyperpigmentation	BCC SCC	Palmoplantar Trunk Upper limb extremities Lower limb extremities	Surgical excision with/without skin flap Radiotherapy
Jackson et al.,^73^ 1975	IV	Case series	*n* = 7	Canada	Keratosis	BCC SCC	Palmoplantar (7) Face Chest Back	Not reported
Khandpur et al.,^50^ 2003	V	Case report	*n* = 1	India	Keratosis	BD SCC	Palmoplantar Upper limb extremities Lower limb extremities Trunk	Acitretin and 5‐fluouracil
Kumar et al.,^54^ 2018	IV	Cross‐sectional	*n* = 39	India	Keratosis Hyperpigmentation Mees' lines	Not reported	Nails of the hands Palmoplantar Generalized	Not reported
Li et al.,^46^ 2016	IV	Case series	*n* = 12	China	Keratosis Hyperpigmentation Hypopigmentation	BD BCC	Scalp Palmoplantar Chest Back Hip	Discontinued exposure
Lien et al.,^56^ 1999	III	Retrospective	*n* = 6	Taiwan	Keratosis Hyperpigmentation Raindrop hypopigmentation	BD SCC MCC	Lip Hands Chest Abdomen	Not reported
Mazumder et al.,^31^ 2009	IV	Cross‐sectional	*n* = 70	Cambodia	Keratosis Hyperpigmentation	Not reported	Unspecified areas	Not reported
Mehta et al.,^74^ 2019	IV	Case series	*n* = 2	India	Keratosis Hyperpigmentation	SCC	Palmoplantar Hand	Not reported
Mukherjee et al.,^13^ 2009	III	Prospective	*n* = 4691	India	Keratosis Hyperpigmentation	BD SCC	Palmoplantar Trunk	Not reported
Ohnishi et al.,^20^ 1997	V	Case report	*n* = 1	Japan	Keratosis Raindrop hyperpigmentation	MCC BD	Palmoplantar Upper limb extremities Trunk	Surgical excision with skin grafts Amputation followed by chemotherapy
Pal et al.,^21^ 2014	V	Case report	*n* = 1	India	Keratosis Raindrop hypopigmentation Ulceration	Not reported	Scalp Generalized	Neosporine ointment and betamethasone valerate Skin graft Empirical antibiotics
Pal et al.,^61^ 2015	IV	Case series	*n* = 1	India	Keratosis Hypopigmentation Hyperpigmentation	SCC	Palmoplantar Upper limb extremities	Not reported
Pratt et al.,^47^ 2016	V	Case report	*n* = 1	Canada	Keratosis	Not reported	Palmoplantar	Discontinued exposure Acitretin Triamcinolone cream
Ramos et al.,^63^ 2008	IV	Case‐control	*n* = 11	Peru	Keratosis Hyperpigmentation Mees' lines	Not reported	Nails of hands Palmoplantar Chest Abdomen	Not reported
Saha et al.,^64^ 2003	III	Retrospective	*n* = 4865	India	Hyperpigmentation	Not reported	Palmoplantar Generalized	Not reported
Singh et al.,^65^ 2013	V	Case report	*n* = 1	Mexico	Keratosis Keratoderma Hyperpigmentation Raindrop hypopigmentation	Not reported	Palmoplantar Abdomen Back	Not reported
Smith et al.,^78^ 2000	IV	Cross‐sectional	*n* = 5	Chile	Keratoses Hyperpigmentation Hypopigmentation	BD	Palmoplantar Upper limb extremities Chest Back	Not reported
Sommers et al.,^33^ 1953	IV	Case series	*n* = 2	Not specified	‐	BD BCC SCC	Fingers Hands Back Lower limb extremities	Amputation Excision Radiation therapy
Sy et al.,^79^ 2017	III	Retrospective	*n* = 116	Philippines	Keratoses Hyperpigmentation	SCC	Palmoplantar Unspecified body parts	Not reported
Tanga et al.,^57^ 2016	IV	Cross sectional	*n* = 46	India	Keratosis	BCC SCC	Palmoplantar Unspecific areas	Surgical excision with/without skin graft, with/without periosteal removal Amputation
Tsuruta et al.,^69^ 1998	V	Case report	*n* = 1	Japan	Keratosis	BD MCC	Fingers Palmoplantar Back	5‐Fluorouracil ointment Surgical excision and skin graft
Verhave et al.,^53^ 2019	V	Case report	*n* = 1	Bangladesh	Keratosis	SCC	Upper limb extremities Trunk Palmoplantar	5‐Fluorouracil cream 5% Nicotinamide Wound care
Walvekar et al.,^54^ 2007	V	Case report	*n* = 1	India	Hyperpigmentation Keratosis	SCC	Scalp Hands Trunk Lower limb extremities	Surgical excision
Watson et al.,^51^ 2004	V	Case report	*n* = 1	Vietnam	Hyperpigmentation Keratosis	BD	Palmoplantar Trunk Upper limb extremities Lower limb extremities	Acitretin
Woollons et al.,^49^ 1998	V	Case report	*n* = 1	Chile	Keratoses Hyperpigmentation	BCC	Palmoplantar Upper limb extremities Trunk	Surgical excision PDT Aquadrate cream
Xia et al.,^74^ 2009	IV	Cross‐sectional	*n* = 632	Mongolia	Keratosis Hyperpigmentation Hypopigmentation	Not reported	Unspecific areas	Not reported
Yerebakan et al.,^23^ 2002	V	Case report	*n* = 2	Turkey	Keratosis Hyperpigmentation Raindrop hypopigmentation	BD SCC	Palmoplantar Trunk Upper limb extremities Lower limb extremities	Acitretin Amputation followed by radiotherapy
Zaldivar et al.,^76^ 1981	IV	Case series	*n* = 2	Chile	Keratosis Hyperpigmentation	SCC	Palmoplantar Trunk	Amputation
Zaldivar et al.,^76^ 1981	IV	Case series	*n* = 16	Chile	Not reported	BCC SCC	Fingers Hand Palmoplantar Chest Trunk Scrotum	Excision

### 4.2 Cutaneous manifestations of acute arsenic toxicity

There are only four articles highlighting cases where individuals had cutaneous presentations along with systemic symptoms associated with acute arsenic poisoning.^7^, ^14^, ^15^, ^16^ As summarized in Table 1, acute arsenic toxicity may be commonly associated with palmoplantar keratosis and lamellar peeling involving nonspecific parts of the body as reported in three of the studies.^7^, ^14^, ^15^ Other clinical presentations such as hyperhidrosis, stomatitis and alopecia were reported once in their respective study but could not be supported by findings in other studies.

**FIGURE 2:**
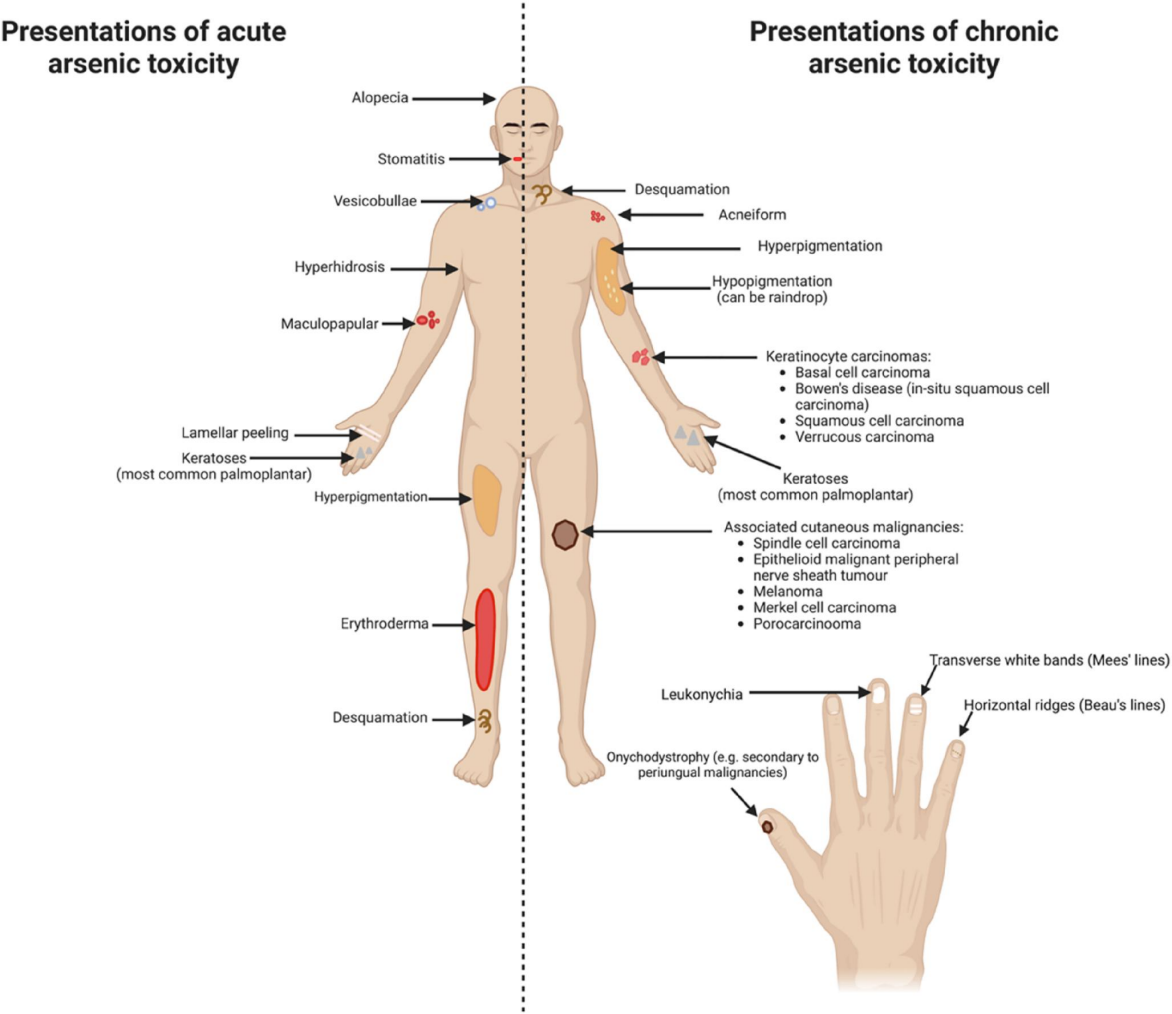
Reported acute and delayed cutaneous manifestations of arsenic toxicity irrespective of criteria established in ‘Synthesis of Findings’.

Two studies, a case report and a retrospective cohort study of 22 participants, suggest chelation therapy with dimercaprol (British anti‐Lewisite [BAL]) may be used in individuals with acute arsenic toxicity.^15^, ^16^ In Tay et al.'s study, 15 individuals with acute arsenic symptoms had effective response with dimercaprol and the participant in Wagner et al.'s case report had resolved symptoms with a 3‐day course of treatment.^15^, ^16^ However, both studies also suggested that although dimercaprol may be effective in acute and subacute cases, cutaneous manifestations of chronic arsenic toxicity can still manifest many years later.^15^, ^16^ Reported cutaneous presentations of acute arsenic poisoning are summarized in Figure 2.

### 4.3 Non‐malignant cutaneous manifestations of chronic arsenic toxicity

Multiple studies selected in this review strongly supports that keratoses, hyper‐ and hypopigmentation can be arsenic‐induced. Hyperpigmentation and hypopigmentation may be generalized or occur as localized patches; a ‘raindrop’ pattern of distribution was commonly reported in exposures to contaminated groundwater and medications.^16^, ^17^, ^18^, ^19^, ^20^, ^21^, ^22^, ^23^ Raindrop patterns occur where there are multiple depigmented macules dispersed against hyperpigmented skin, or when multiple pigmented macules are present against normal skin.^16^ Specific areas where pigmented patches are confined include the face, upper and lower limb extremities or truncal regions. Pigmented regions may appear in various shades of brown and can be diffuse, reticulate or homogenously localized.^24^ Several differentials for melanosis include freckles, lentigines, post‐inflammatory hyperpigmentation, macular lichen planus and ashy melanosis.^25^ Differential diagnoses for hypopigmentation include pityriasis versicolour, post‐inflammatory hypopigmentation, idiopathic guttate hypomelanosis, leprosy and salt‐and‐pepper pigmentation of systemic sclerosis.^25^

Keratoses were the second most commonly reported presentation of chronic arsenic poisoning and may occur in isolation or accompanying pigmentary changes.^16^ Arsenic keratoses may present as multiple, small, discrete, spiny or wart‐like papules. These have a predilection for sun‐exposed sites but may occur anywhere. Acrally based keratoses are more often observed on palms and soles rather than dorsal surfaces.^26^, ^27^, ^28^, ^29^, ^30^ With increasing severity, arsenical keratoses may present as Bowenoid plaques and cutaneous horns.^31^ Punctate, follicular, band‐like and crateriform morphologies have also been described.^24^, ^32^ Reported cutaneous presentations are summarized in Figure 2. Various differentials to consider for arsenical keratoses include verruca vulgaris, seborrhoeic keratosis, lichen amyloidosis, hypertrophic lichen planus, occupational keratosis, punctate palmoplantar keratoderma and punctate porokeratosis.^25^, ^33^

Nail changes were noted in seven studies consisting of case reports, retrospective cohort and cross‐sectional studies.^14^, ^16^, ^17^, ^27^, ^34^, ^35^ Mees' lines were consistently reported in all seven studies with Uede et al. being the only study to highlight other features such as complete leukonychia, Beau's lines and onychodystrophy.^14^

### 4.4 Malignant cutaneous manifestations of chronic arsenic toxicity

Whilst chronic arsenic exposure may be associated with BCC, SCC and BD, there may be rare associated malignancies to consider (Figure 2). Five studies, consisting a combination of case reports and retrospective studies suggested an association between arsenic exposure and Merkel cell carcinoma (MCC).^12^, ^19^, ^20^, ^36^, ^37^ These cases had a predilection for the trunk and acral sites which is contrary to classical cases which more commonly arise on the head.^19^, ^20^, ^36^

Other rare cutaneous tumours were identified in case reports of suspected arsenic exposure but not in other selected articles with higher level of evidence. Cases included reports of porocarcinoma, dermatofibrosarcoma protuberans, hidradenocarcinoma and epithelioid malignant peripheral nerve sheath tumour (EMPNST).^9^, ^10^, ^38^, ^39^, ^40^

### 4.5 Treatment of chronic arsenic toxicity dermatological presentations

Multiple studies have demonstrated that eliminating arsenic exposure is strongly associated with preventing further cutaneous manifestations.^5^, ^41^, ^42^, ^43^, ^44^, ^45^, ^46^, ^47^ Although both Tay et al. and Wagner et al.'s findings suggested dimercaprol may only be effective in acute and subacute arsenic toxicity, other chelation therapy may improve some dermatological presentations in the chronic phase.^15^, ^16^ A case report and case series demonstrated a positive association between individuals taking oral D‐penicillamine with improvement of cutaneous presentations such as palmoplantar hyperkeratosis that were suspected to be chronic arsenic‐induced.^17^, ^44^ Zhou et al. also reported that sodium dimercaptosulfonate, a less toxic BAL salt, given as two courses of 0.25 mg oral daily for 3 days each can improve pruritus and arsenic‐induced keratoses.^34^ Other case reports noted the use of 5%–10% salicylic acids in reducing hyperkeratosis suspected to be caused by chronic arsenic exposure.^27^, ^48^, ^49^ Five case reports suggested that in presentations where there may be hyperkeratosis with concurrent cutaneous malignancies, individuals may respond well with oral retinoids such as acitretin, dose ranged 10–25 mg/day, in combination with other management strategies.^23^, ^27^, ^47^, ^50^, ^51^

Multiple studies of variable level of evidence suggests that standard management of keratinocyte carcinomas may still be effective in those with chronic arsenicism. These included: topical 5‐fluorouracil, imiquimod, cryotherapy, Mohs micrographic surgery, and conventional excision with/without skin graft.^5^, ^9^, ^12^, ^15^, ^16^, ^18^, ^36^, ^37^, ^39^, ^52^, ^53^, ^54^, ^55^ Radiotherapy was also reported to be used as first line treatment as well as adjuvant therapy.^23^, ^36^, ^39^, ^52^ Meanwhile, amputations have been used to manage advanced SCC and MCC.^20^, ^39^, ^56^, ^57^ Mix case reports and series showed that photodynamic therapy and carbon dioxide laser may be effective in eliminating superficial cutaneous premalignant and malignant conditions in cases with suspected chronic arsenic toxicity.^11^, ^49^, ^58^ Three case reports identified authors prescribing nicotinamide to prevent further development of non‐melanocytic malignancies after treatment of arsenic‐induced keratinocyte carcinomas, however this was not reported in studies of higher level of evidence.^5^, ^53^, ^59^

## 5 DISCUSSION

This systematic review aimed to identify cutaneous manifestations of arsenic toxicity and its management in adults as well as to critically appraise current literature. There were variable cutaneous presentations in those with acute arsenic toxicity, thus making it challenging to identify associated link. Common cutaneous manifestations that were strongly associated with chronic arsenic toxicity include hyperpigmentation, hypopigmentation and keratosis and are often present in addition to keratinocyte neoplasms as highlighted in extensive studies consisting of case reports, retrospective and cross‐sectional studies of large sample population size.

Although rare tumours were reported in case reports, their association with arsenic exposure remains inconclusive. Individuals with these rare carcinomas had a history of previous arsenic exposure, but causation could not be proven and some case reports did not have objective measurements such as arsenic levels in sources or samples obtained from those affected to confirm their suspicion based on history. Further reports may be limited by the rarity of these carcinomas, and conducting studies of higher level of evidence is not ethically possible.

Avoiding further exposure is a common management of chronic arsenic toxicity in several studies. Low level evidence studies suggest that chelation therapy including dimercaprol, sodium dimercaptosulfonate and D‐penicillamine may be potential treatments.^15^, ^16^, ^17^, ^34^, ^44^ Dimercaptosuccinic acid is also another arsenic chelator, but none of the selected studies demonstrated its use in treating arsenic toxicities for those with cutaneous presentations.^17^ There were vast therapeutic options reported for arsenic‐associated hyperkeratosis and common keratinocyte carcinomas, with treatments likely guided by specialists' clinical judgement and local guidelines.

Challenges to identify causation between arsenic exposure and cutaneous manifestations are due to how individuals were exposed to arsenic. Individuals diagnosed in developed nations may have been environmentally exposed in endemic countries at a younger age.^42^, ^47^, ^55^, ^59^ Those with presentations likely attributed to environmental exposure in endemic countries, such as from drinking contaminated groundwater, are often in the older aged group with the time of exposure being decades prior to presentation and thus a causative link can never be proven.^40^, ^51^, ^52^ Similarly, individuals with occupational exposures to arsenic may be of variable age at time of cutaneous manifestations, however, the time between exposure and presentation may be months to years depending on the frequency, route and cumulative dose of arsenic.^23^, ^60^, ^61^ Occupations where arsenic exposure may occur include carpentry, pesticide application in agriculture, non‐ferrous metal mining and smelting, coal or wood combustion and manufacturing of computer microchips.^42^, ^55^

Diagnoses of arsenic toxicity are likely to be supported if mode of exposure is through ingestion of traditional and Western medicines, as cutaneous presentations may manifest acutely with other systemic symptoms due to incorrect compounding.^41^ In developed countries, arsenic is still found in the treatment of acute promyelocytic leukemia as well in traditional medicines such as Chinese herbal medicines, Indian Ayurvedic medicines and homoeopathic remedies.^42^, ^58^ As arsenic‐containing medicines are used to treat chronic disorders with variable compounded concentrations, associations may be weakly inferred but establishing causation is challenging given that there are other external factors that may contribute to sporadic manifestations such as keratinocyte carcinomas.^5^, ^6^, ^28^, ^62^, ^63^, ^64^

There are various methods to estimate arsenic levels in various samples. Currently, the ‘gold standard’ is atomic absorption spectrometry due to its high specificity and sensitivity compared to other methods.^25^ Hairs are collected after washing with arsenic‐free shampoo and nail samples are collected by clipping all fingernails and toenails that have grown for a month.^2^ Diagnosis of arsenic toxicity is strongly suggested if arsenic level is greater than 1 mg/kg of dry hair and greater than 1.5 mg/kg of nails.^2^ Water and urine samples are collected in acid‐washed plastic containers, with addition of concentrated hydrochloric acid in urine samples to prevent bacterial growth. Arsenic levels greater than 50 μg/L in water and urine samples are strongly suggestive of arsenic toxicity. While no definitive biomarkers are identified, studies have suggested that elevated levels of urinary uroporphyrin‐III and coproporphyrin‐III or low serum levels of metallothionein may be potential markers of arsenic toxicity.^65^, ^66^

The strength of this systematic review is that selected studies also had wide representation of timespan (1953–2019) and representation of developed and developing countries where cases are reported in regards to type of exposure, which is particularly important for those affected by environmental or occupational exposure. There are also several limitations to the review. Multiple low level of evidence studies reported dermatological findings but its link with suspected arsenic exposure were only inferred based on history. Subsequently, the majority of these studies lack causal relationships but there were stronger associations in higher level of evidence studies. This also extends to management strategies where treatments for some clinical presentations were reported, but the evidence for its efficacy in context of suspected arsenic toxicity may be low due to lack of reporting in interventional studies or high level of evidence articles.

## 6 CONCLUSIONS

There is a broad spectrum of dermatological findings for acute and chronic arsenic toxicity reported in current literature. Common stigmata of chronic arsenic toxicity including keratoses and pigmentary changes, as well as potential concurrent keratinocyte tumours. However, uncommon manifestations reported are only inferred based on history to be weakly associated with arsenic exposure due to multiple low‐level evidence studies identified, and treatment efficacy reported are not supported by studies of higher level of evidence.

## CONFLICT OF INTEREST STATEMENT

All authors involved in this work have no conflicts of interest to disclose.

## AUTHOR CONTRIBUTIONS

**Gia Toan Tang**: Data curation (Equal); Formal analysis (Equal); Investigation (Equal); Methodology (Equal); Software (Equal); Visualization (Equal); Writing – original draft (Equal); Writing – review & editing (Equal). **Joshua Elakis**: Formal analysis (Equal); Investigation (Equal); Methodology (Equal); Supervision (Equal); Writing – original draft (Equal); Writing – review & editing (Equal). **Laura Scardamaglia**: Conceptualization (Equal); Methodology (Equal); Supervision (Equal); Writing – review & editing (Equal).

## ETHICS STATEMENT

Not applicable.

## Supporting information

Supporting Information S1

## Data Availability

The data that supports the findings of this study are available in the supplementary material of this article.
