# Supplementary material for: Cutaneous manifestations and treatment of arsenic toxicity: A systematic review
Source: Skin Health Dis. 2023 Mar 25;3(4):e231. doi: 10.1002/ski2.231 (PMC10395639; doi:10.1002/ski2.231)
Supplement: Supplementary file 1 — Supporting Information S1 [file SKI2-3-e231-s001.docx]

Table S1: Summary of consensus response for JBI checklist critical appraisal tools for case reports

| Study ID | Were patient's demographic characteristics clearly described? | Was the patient's history clearly described and presented as a timeline? | Was the current clinical condition of the patient on presentation clearly described? | Were diagnostic tests or assessment methods and the results clearly described? | Was the intervention(s) or treatment procedure(s) clearly described? | Was the post-intervention clinical condition clearly described? | Were adverse events (harms) or unanticipated events identified and described? | Does the case report provide takeaway lessons? | Overall Appraisal |
| --- | --- | --- | --- | --- | --- | --- | --- | --- | --- |
| Pal *et al*., 2015 | Yes | Yes | Yes | Unclear | No | No | Not applicable | Unclear | Include |
| Pinto *et al*., 2014 | Yes | Yes | Yes | Yes | Yes | Yes | No | Yes | Include |
| Chen *et al*., 2017 | Yes | Yes | Yes | Yes | Yes | Yes | No | Yes | Include |
| Pollo *et al*., 2019 | Yes | Yes | Yes | Yes | Yes | Yes | No | Yes | Include |
| Verhave *et al*., 2019 | Yes | No | Yes | Yes | Yes | Yes | No | Unclear | Include |
| Ding *et al*., 2018 | Yes | Yes | Yes | Yes | Yes | Yes | Not applicable | Yes | Include |
| Hinojosa *et al*., 2018 | Yes | Yes | Yes | Yes | Unclear | No | Not applicable | Unclear | Include |
| Pratt *et al*., 2015 | Yes | Yes | Yes | Yes | Yes | No | No | Yes | Include |
| Kim *et al*., 2013 | Yes | Yes | Yes | Yes | Yes | No | Not applicable | Unclear | Include |
| Singh *et al*., 2013 | Yes | Yes | Yes | Yes | Not applicable | Not applicable | Not applicable | Unclear | Include |
| Gulshan *et al*., 2016 | Yes | Yes | Yes | Yes | Yes | Yes | Not applicable | Unclear | Include |
| Chou *et al*., 2016 | Yes | No | Yes | Yes | Yes | Yes | Yes | Yes | Include |
| Lonergan *et al*., 2010 | Yes | Not applicable | Yes | Unclear | Yes | Yes | Not applicable | Yes | Include |
| Zhou *et al*., 2015 | Yes | Yes | Yes | Yes | Yes | Yes | Yes | Yes | Include |
| Ramirez *et al*., 2012 | Unclear | Yes | No | Yes | Yes | No | No | Yes | Include |
| Zhu *et al*., 2012 | Yes | Yes | Yes | Yes | No | Not applicable | Yes | Yes | Include |
| Watson *et al*., 2004 | Yes | No | Yes | Yes | Yes | Yes | Yes | No | Include |
| Murata *et al*., 1994 | Yes | Yes | Yes | Yes | Not applicable | Yes | Not applicable | Yes | Include |
| Hanjani *et al*., 2007 | Yes | Yes | Yes | Yes | Yes | Yes | Not applicable | Yes | Include |
| Gerdsen *et al*., 2000 | Yes | No | Yes | Yes | Yes | Unclear | Not applicable | Unclear | Include |
| Col *et al*., 1999 | Yes | Yes | Yes | Yes | Yes | Yes | No | Yes | Include |
| Bartolome *et al*., 1999 | Yes | Yes | Yes | No | Yes | Yes | Not applicable | Yes | Include |
| Kim *et al*., 1999 | Yes | Yes | Yes | Yes | Yes | No | Not applicable | Unclear | Include |
| Woollons *et al*., 1998 | Yes | Yes | Yes | Yes | No | No | No | Yes | Include |
| Tsuruta *et al*., 1998 | Yes | Yes | Yes | Yes | Not applicable | Not applicable | Not applicable | Unclear | Include |
| Ohnishi *et al*., 1997 | Yes | Yes | Yes | Yes | Yes | Yes | Yes | Yes | Include |
| Chen *et al*., 2005 | Yes | No | Yes | Yes | Yes | Yes | No | Yes | Include |
| Ho *et al*., 2005 | Yes | Unclear | Yes | Yes | Unclear | Yes | Not applicable | Yes | Include |
| Khandpur *et al*., 2003 | Yes | Yes | Yes | Yes | Yes | Yes | No | Yes | Include |
| Park *et al*., 2002 | Yes | Yes | Yes | Yes | Yes | Yes | No | Yes | Include |
| Yerebakan *et al*., 2002 | Yes | Yes | Yes | Yes | Yes | Yes | Yes | Yes | Include |
| Southwick *et al*., 1979 | Yes | Yes | Yes | Yes | Yes | Yes | Yes | Yes | Seek further Info |
| Wagner *et al*., 1979 | Yes | Yes | Yes | Yes | Unclear | Unclear | Yes | Yes | Include |
| Shneidman *et al*., 1986 | Yes | Yes | Yes | Yes | Yes | No | No | Unclear | Seek further Info |
| Kaur *et al*., 1982 | Yes | Yes | Yes | Yes | No | No | No | Unclear | Include |
| HILL *et al*., 1964 | Yes | Yes | Yes | Unclear | Yes | Yes | Not applicable | Unclear | Include |
| Siefring *et al*., 2018 | Yes | Yes | Yes | Yes | Yes | Yes | Yes | Yes | Include |
| Pal *et al*., 2014 | Yes | Yes | Yes | Yes | Unclear | Unclear | No | Unclear | Include |
| Das *et al*., 2012 | Yes | Yes | Yes | Yes | Yes | No | Not applicable | Yes | Include |
| Walvekar *et al*., 2007 | Yes | Yes | Yes | Yes | Yes | Yes | Yes | Yes | Include |
| Sass *et al*., 1993 | Yes | Yes | No | Unclear | Yes | Yes | Yes | Unclear | Include |
| Seok *et al*., 2015 | Yes | Yes | Yes | Yes | Yes | No | No | Yes | Include |

Table S2: Summary of consensus response for JBI checklist critical appraisal tools for case series

| Study ID | Were there clear criteria for inclusion in the case series? | Was the condition measured in a standard, reliable way for all participants included in the case series? | Were valid methods used for identification of the condition for all participants included in the case series? | Did the case series have consecutive inclusion of participants? | Did the case series have complete inclusion of participants? | Was there clear reporting of the demographics of the participants in the study? | Was there clear reporting of clinical information of the participants? | Were the outcomes or follow up results of cases clearly reported? | Was there clear reporting of the presenting site(s)/clinic(s) demographic information? | Was statistical analysis appropriate? | Overall Appraisal |
| --- | --- | --- | --- | --- | --- | --- | --- | --- | --- | --- | --- |
| Ahsan *et al*., 2009 | No | Yes | Yes | Yes | Yes | Yes | Yes | No | No | Not applicable | Include |
| Zaldivar *et al*., 1981 | Yes | No | Unclear | Unclear | Unclear | Yes | Yes | No | Unclear | Not applicable | Include |
| Cabrera *et al*., 2003 | Yes | Yes | Yes | Yes | Yes | Yes | Unclear | No | Yes | Yes | Include |
| Li *et al*., 2016 | Yes | Yes | Yes | Not applicable | Yes | Yes | Yes | No | Yes | Not applicable | Include |
| Jaafar *et al*., 1993 | Not applicable | Unclear | Unclear | Unclear | Unclear | Yes | Yes | Yes | Yes | Not applicable | Include |
| Tantikun *et al*., 2000 | Yes | Yes | Yes | Yes | Yes | Yes | Yes | Yes | Unclear | Not applicable | Include |
| Lien *et al*., 1999 | Unclear | Not applicable | Yes | Not applicable | Yes | Yes | Yes | No | Yes | Not applicable | Include |
| Wong *et al*., 1998 | No | Unclear | Unclear | Unclear | Unclear | Yes | Yes | Yes | Unclear | Not applicable | Include |
| Centeno *et al*., 2002 | Yes | Yes | Yes | No | Not applicable | Yes | Yes | No | Yes | Not applicable | Include |
| Sommers *et al*., 1953 | No | Unclear | Unclear | Unclear | Unclear | Yes | Yes | Yes | Not applicable | Not applicable | Include |
| Mehta *et al*., 2019 | No | No | No | No | Yes | Yes | Yes | No | Yes | Not applicable | Seek further Info |
| SaiSiongWong *et al*., 1998 | Yes | Yes | Yes | Not applicable | Yes | Yes | Yes | No | Yes | Yes | Include |
| Jackson *et al*., 1975 | Yes | Unclear | Unclear | Yes | Yes | Yes | Yes | Yes | Unclear | Not applicable | Include |
| Tay *et al*., 1974 | Yes | Unclear | Yes | Yes | Yes | Unclear | Yes | Yes | Unclear | Not applicable | Include |
| Saha *et al*., 2003 | No | Yes | Unclear | Unclear | Unclear | No | Yes | No | No | Not applicable | Include |
| Chakraborti *et al*., 2003 | No | Yes | Yes | Yes | Yes | Yes | Yes | No | Yes | Not applicable | Include |
| Ghosh *et al*., 2013 | Yes | Yes | Yes | Yes | Yes | No | Yes | No | Yes | Yes | Include |

Table S3: Summary of consensus response for JBI checklist critical appraisal tools for case control studies

| Study ID | Were the groups comparable other than the presence of disease in cases or the absence of disease in controls? | Were cases and controls matched appropriately? | Were the same criteria used for identification of cases and controls? | Was exposure measured in a standard, valid and reliable way? | Was exposure measured in the same way for cases and controls? | Were confounding factors identified? | Were strategies to deal with confounding factors stated? | Were outcomes assessed in a standard, valid and reliable way for cases and controls? | Was the exposure period of interest long enough to be meaningful? | Was appropriate statistical analysis used? | Overall appraisal |
| --- | --- | --- | --- | --- | --- | --- | --- | --- | --- | --- | --- |
| Ghosh *et al*., 2013 | Unclear | No | No | Yes | Yes | No | No | Yes | Yes | Yes | Include |
| Ramos *et al*., 2008 | Yes | Unclear | Yes | Unclear | Yes | Not applicable | Not applicable | Yes | Yes | Yes | Seek further Info |

Table S4: Summary of consensus response for JBI checklist critical appraisal tools for cohort studies

| Study ID | Were the two groups similar and recruited from the same population? | Were the exposures measured similarly to assign people to both exposed and unexposed groups? | Was the exposure measured in a valid and reliable way? | Were confounding factors identified? | Were strategies to deal with confounding factors stated? | Were the groups/participants free of the outcome at the start of the study (or at the moment of exposure)? | Were the outcomes measured in a valid and reliable way? | Was the follow up time reported and sufficient to be long enough for outcomes to occur? | Was follow up complete, and if not, were the reasons to loss to follow up described and explored? | Were strategies to address incomplete follow up utilized? | Was appropriate statistical analysis used? | Overall appraisal |
| --- | --- | --- | --- | --- | --- | --- | --- | --- | --- | --- | --- | --- |
| Choudhury *et al*., 2018 | Not applicable | Not applicable | Yes | No | Not applicable | Yes | Yes | Yes | Yes | Not applicable | Yes | Include |
| Uede *et al*., 2003 | Not applicable | Not applicable | Unclear | No | Not applicable | Yes | Yes | Yes | No | No | Yes | Include |

Table S5: Summary of consensus response for JBI checklist critical appraisal tools for cross sectional studies

| Study ID | Were the criteria for inclusion in the sample clearly defined? | Were the study subjects and the setting described in detail? | Was the exposure measured in a valid and reliable way? | Were objective, standard criteria used for measurement of the condition? | Were confounding factors identified? | Were strategies to deal with confounding factors stated? | Were the outcomes measured in a valid and reliable way? | Was appropriate statistical analysis used? | Overall |
| --- | --- | --- | --- | --- | --- | --- | --- | --- | --- |
| Mazumder *et al*., 2009 | Yes | Yes | Yes | Unclear | Yes | Unclear | Unclear | Not applicable | Include |
| Ishinishi *et al*., 1977 | Yes | Yes | Yes | Unclear | No | No | Yes | Unclear | Seek further Info |
| Sy *et al*., 2017 | Yes | Yes | Not applicable | Yes | Yes | Yes | Yes | Not applicable | Include |
| Tanga *et al*., 2016 | Yes | Unclear | Unclear | Yes | No | Not applicable | Unclear | Not applicable | Include |
| Xia *et al*., 2009 | Yes | Yes | Yes | Unclear | Yes | Yes | Unclear | Yes | Include |
| Smith *et al*., 2000 | Yes | Yes | Unclear | Unclear | Yes | Yes | Yes | Not applicable | Include |
| AhmadSk *et al*., 1999 | Unclear | Yes | Yes | Yes | Not applicable | Not applicable | Yes | Yes | Include |
| Mukherjee *et al*., 2005 | Unclear | Yes | Yes | Unclear | No | Not applicable | Unclear | Yes | Include |
| Kumar *et al*., 2018 | Yes | Yes | Yes | Yes | No | Not applicable | Yes | Yes | Include |

Table S6: Table summary of percentage of total responses for each JBI checklist critical appraisal tool for case reports

|  | Yes% | No% | Unclear% | Not applicable% |
| --- | --- | --- | --- | --- |
| Were patient's demographic characteristics clearly described? | 97.61905 | 0 | 2.380952 | 0 |
| Was the patient's history clearly described and presented as a timeline? | 83.33333 | 11.90476 | 2.380952 | 2.380952 |
| Was the current clinical condition of the patient on presentation clearly described? | 95.2381 | 4.761905 | 0 | 0 |
| Were diagnostic tests or assessment methods and the results clearly described? | 88.09524 | 2.380952 | 9.52381 | 0 |
| Was the intervention(s) or treatment procedure(s) clearly described? | 73.80952 | 9.52381 | 9.52381 | 7.142857 |
| Was the post-intervention clinical condition clearly described? | 59.52381 | 26.19048 | 7.142857 | 7.142857 |
| Were adverse events (harms) or unanticipated events identified and described? | 26.19048 | 35.71429 | 0 | 38.09524 |
| Does the case report provide takeaway lessons? | 64.28571 | 2.380952 | 33.33333 | 0 |

Table S7: Table summary of percentage of total responses for each JBI checklist critical appraisal tool for case series

|  | Yes% | No% | Unclear% | Not applicable% |
| --- | --- | --- | --- | --- |
| Were there clear criteria for inclusion in the case series? | 52.94118 | 35.29412 | 5.882353 | 5.882353 |
| Was the condition measured in a standard, reliable way for all participants included in the case series? | 52.94118 | 11.76471 | 29.41176 | 5.882353 |
| Were valid methods used for identification of the condition for all participants included in the case series? | 58.82353 | 5.882353 | 35.29412 | 0 |
| Did the case series have consecutive inclusion of participants? | 41.17647 | 11.76471 | 29.41176 | 17.64706 |
| Did the case series have complete inclusion of participants? | 64.70588 | 0 | 29.41176 | 5.882353 |
| Was there clear reporting of the demographics of the participants in the study? | 82.35294 | 11.76471 | 5.882353 | 0 |
| Was there clear reporting of clinical information of the participants? | 94.11765 | 0 | 5.882353 | 0 |
| Were the outcomes or follow up results of cases clearly reported? | 35.29412 | 64.70588 | 0 | 0 |
| Was there clear reporting of the presenting site(s)/clinic(s) demographic information? | 52.94118 | 11.76471 | 29.41176 | 5.882353 |
| Was statistical analysis appropriate? | 17.64706 | 0 | 0 | 82.35294 |

Table S8: Table summary of percentage of total responses for each JBI checklist critical appraisal tool for case control studies

|  | Yes% | No% | Unclear% | Not applicable% |
| --- | --- | --- | --- | --- |
| Were the groups comparable other than the presence of disease in cases or the absence of disease in controls? | 50 | 0 | 50 | 0 |
| Were cases and controls matched appropriately? | 0 | 50 | 50 | 0 |
| Were the same criteria used for identification of cases and controls? | 50 | 50 | 0 | 0 |
| Was exposure measured in a standard, valid and reliable way? | 50 | 0 | 50 | 0 |
| Was exposure measured in the same way for cases and controls? | 100 | 0 | 0 | 0 |
| Were confounding factors identified? | 0 | 50 | 0 | 50 |
| Were strategies to deal with confounding factors stated? | 0 | 50 | 0 | 50 |
| Were outcomes assessed in a standard, valid and reliable way for cases and controls? | 100 | 0 | 0 | 0 |
| Was the exposure period of interest long enough to be meaningful? | 100 | 0 | 0 | 0 |
| Was appropriate statistical analysis used? | 100 | 0 | 0 | 0 |

Table S9: Table summary of percentage of total responses for each JBI checklist critical appraisal tool for cohort studies

|  | Yes% | No% | Unclear% | Not applicable% |
| --- | --- | --- | --- | --- |
| Were the two groups similar and recruited from the same population? | 0 | 0 | 0 | 100 |
| Were the exposures measured similarly to assign people to both exposed and unexposed groups? | 0 | 0 | 0 | 100 |
| Was the exposure measured in a valid and reliable way? | 50 | 0 | 50 | 0 |
| Were confounding factors identified? | 0 | 100 | 0 | 0 |
| Were strategies to deal with confounding factors stated? | 0 | 0 | 0 | 100 |
| Were the groups/participants free of the outcome at the start of the study (or at the moment of exposure)? | 100 | 0 | 0 | 0 |
| Were the outcomes measured in a valid and reliable way? | 100 | 0 | 0 | 0 |
| Was the follow up time reported and sufficient to be long enough for outcomes to occur? | 100 | 0 | 0 | 0 |
| Was follow up complete, and if not, were the reasons to loss to follow up described and explored? | 50 | 50 | 0 | 0 |
| Were strategies to address incomplete follow up utilized? | 0 | 50 | 0 | 50 |
| Was appropriate statistical analysis used? | 100 | 0 | 0 | 0 |

Table S10: Table summary of percentage of total responses for each JBI checklist critical appraisal tool for cross sectional studies

|  | Yes% | No% | Unclear% | Not applicable% |
| --- | --- | --- | --- | --- |
| Were the criteria for inclusion in the sample clearly defined? | 77.77778 | 0 | 22.22222 | 0 |
| Were the study subjects and the setting described in detail? | 88.88889 | 0 | 11.11111 | 0 |
| Was the exposure measured in a valid and reliable way? | 66.66667 | 0 | 22.22222 | 11.11111 |
| Were objective, standard criteria used for measurement of the condition? | 44.44444 | 0 | 55.55556 | 0 |
| Were confounding factors identified? | 44.44444 | 44.44444 | 0 | 11.11111 |
| Were strategies to deal with confounding factors stated? | 33.33333 | 11.11111 | 11.11111 | 44.44444 |
| Were the outcomes measured in a valid and reliable way? | 55.55556 | 0 | 44.44444 | 0 |
| Was appropriate statistical analysis used? | 44.44444 | 0 | 11.11111 | 44.44444 |

Table S11: Table summary of percentage of overall appraisal responses

| Study type | Include% | Seek Further Info% |
| --- | --- | --- |
| Case report | 95.23809524 | 4.761904762 |
| Case series | 94.11765 | 5.882353 |
| Case control | 50 | 50 |
| Cohort studies | 100 | 0 |
| Cross sectional studies | 88.88889 | 11.11111 |
